# Neuroinflammation at the Neuroforamina and Spinal Cord in Patients with Painful Cervical Radiculopathy and Pain-Free Participants: An [^11^C]DPA713 PET/CT Proof-of-Concept Study

**DOI:** 10.3390/jcm14072420

**Published:** 2025-04-02

**Authors:** Ivo J. Lutke Schipholt, Meghan A. Koop, Michel W. Coppieters, Elsmarieke M. van de Giessen, Adriaan A. Lammerstma, Bastiaan C. ter Meulen, Carmen Vleggeert-Lankamp, Bart N.M. van Berckel, Joost Bot, Hans van Helvoirt, Paul R. Depauw, Ronald Boellaard, Maqsood Yaqub, Gwendolyne Scholten-Peeters

**Affiliations:** 1 Department of Human Movement Sciences, Faculty of Behavioural and Movement Sciences, Vrije Universiteit Amsterdam, Amsterdam Movement Sciences—Program Musculoskeletal Health, Van der Boechorststraat 9, 1081 BT Amsterdam, The Netherlands; i.j.m.lutkeschipholt@vu.nl (I.J.L.S.);; 2 Laboratory Medical Immunology, Department of Clinical Chemistry, Amsterdam University Medical Centre, Location VUmc, 1081 HV Amsterdam, The Netherlands; 3 School of Health Sciences and Social Work, Griffith University, Brisbane 4215, Australia; 4 Radiology & Nuclear Medicine, Amsterdam UMC, Location VUmc, 1081 HV Amsterdam, The Netherlands; 5 Department of Radiology & Nuclear Medicine, Amsterdam UMC, Location University of Amsterdam, 1105 AZ Amsterdam, The Netherlands; 6 Department of Nuclear Medicine and Molecular Imaging, University of Groningen, University Medical Center Groningen, 9713 GZ Groningen, The Netherlands; 7 Department of Neurology, OLVG Amsterdam, 1091 AC Amsterdam, The Netherlands; 8 Department of Epidemiology and Biostatistics Amsterdam Movement Sciences Research Institute, Amsterdam UMC, Vrije Universiteit Amsterdam, 9713 GZ Amsterdam, The Netherlands; 9 Department of Neurosurgery, Leiden University Medical Center, 2333 ZG Leiden, The Netherlands; 10 Stichting Rugpoli, 7491 HP Delden, The Netherlands; 11 Department of Neurosurgery, Elisabeth-TweeSteden Ziekenhuis, 5022 GC Tilburg, The Netherlands

**Keywords:** neuroinflammation, neuropathic pain, PET imaging, cervical radiculopathy, immunoactivation

## Abstract

**Background/Objectives:** The complex pathophysiology of painful cervical radiculopathy is only partially understood. Neuroimmune activation in the dorsal root ganglion and spinal cord is assumed to underlie the genesis of radicular pain. Molecular positron emission tomography (PET) using the radiotracer [^11^C]DPA713, which targets the 18-kDa translocator protein (TSPO), offers the ability to quantify neuroinflammation in humans in vivo. The primary objectives of this study were to (1) assess whether uptake of [^11^C]DPA713, a metric of neuroinflammation, is higher in the neuroforamina and spinal cord of patients with painful cervical radiculopathy compared with that in pain-free participants and (2) assess whether [^11^C]DPA713 uptake is associated with clinical parameters, such as pain intensity. **Methods:** Dynamic 60 min [^11^C]DPA713 PET/CT scans were acquired, and regions of interest were defined for neuroforamina and spinal cord. Resulting time-activity curves were fitted to a single-tissue compartment model using an image-derived input function, corrected for plasma-to-whole blood ratios and parent fractions, to obtain the volume of distribution (*V*_T_) as the primary outcome measure. Secondary neuroinflammation metrics included 1T2k *V*_T_ without metabolite correction (1T2k_WB) and Logan *V*_T_. **Results:** The results indicated elevated levels of 1T2k *V*_T_ at the neuroforamina (*p* < 0.04) but not at the spinal cord (*p* = 0.16). Neuroforamina and spinal cord 1T2k *V*_T_ lack associations with clinical parameters. Secondary neuroinflammatory metrics show associations with clinical parameters such as the likelihood of neuropathic pain. **Conclusions:** These findings enhance our understanding of painful cervical radiculopathy’s pathophysiology, emphasizing the neuroforamina levels of neuroinflammation as a potential therapeutic target.

## 1. Introduction

Painful cervical radiculopathy causes significant health issues affecting physical, mental, and social well-being [1]. The pathophysiology is assumed to involve irritation of the cervical nerve roots and/or dorsal root ganglion (DRG) due to compression or chemical cascades. Clinical features of painful cervical radiculopathy include radicular pain with neurological deficits such as sensory loss and weakness [2]. These clinical features arise due to chemical cascades, ischemia, and/or mechanical deformation of neural structures [3,4,5,6,7,8].

Chronic compression of the dorsal root ganglion (CCD) and chronic constriction injury (CCI) are commonly used animal models for studying neuropathic pain caused by peripheral compression neuropathy [9,10,11,12,13]. Preclinical studies indicate that the disturbed homeostasis triggers infiltration of inflammatory cells into affected areas and DRG, activating resident immune cells and producing inflammatory mediators [14,15]. Interaction between mast cells, neutrophils, T-cells, microglia, and astrocytes with neuronal cells induces neuroinflammation and contributes to the development of pain by releasing pro-inflammatory cytokines (e.g., TNF, IL-1β, and reactive oxygen species) that sensitize nociceptive pathways. These neuroimmune interactions lead to intracellular signaling cascades that result in an increase in membrane excitability, a change in receptor expression (e.g., TSPO on glial cells), synaptic efficacy, and/or reduced inhibition of neurons and circuits in nociceptive processing pathways [16,17,18]. This neuroinflammation also occurs in the ventral horn of the spinal cord and in supraspinal regions such as the midbrain, a process called remote immune-mediated neuroinflammation [11,19].

CCD and CCI induce severe nerve compression and can result in significant neuronal damage, such as axon loss, and provoke pronounced neuroinflammation [9,10,11,12,13]. Conversely, progressive mild nerve compression primarily affects small-diameter but not large-diameter axons [19]. In addition, mild compression results in local and remote immune-mediated neuroinflammation depending on the degree of compression [19]. The triggers for activation of spinal neuroinflammation are thought to be substances released by activity in the terminals of damaged afferent neurons and/or aberrant neural activity (neurogenic neuroinflammation) [20]. However, the damage induced by mild compression is probably not severe enough to cause spinal neuroinflammation, a characteristic of many other experimental models of neuropathic pain [11,19,21]. The clinical translation of immune-mediated neuroinflammation observed in preclinical models with severe nerve compression to conditions like painful cervical radiculopathy with milder compression is mostly lacking and requires further studies. Due to ethical and invasive constraints, obtaining information about neuroinflammation in the DRG and spinal cord in humans through immunohistological methods is not feasible. However, positron emission tomography (PET) imaging offers a non-invasive, in vivo alternative by assessing receptor-ligand binding interactions as a proxy for neuroinflammation.

Microglia, expressing the translocator protein (TSPO), play an important role in neuroinflammation and can be measured in vivo using PET with specific radiotracers [22,23]. A study using [^11^C]PBR28 PET/MR in patients with lumbar radiculopathy revealed elevated TSPO levels (standardized uptake value (SUV)) in neuroforamina and spinal cord [24]. A small PET/MR study using [¹¹C]DPA713 (*n* = 5) found no elevated SUV levels at the spinal cord in patients with cervical radiculopathy [25]. However, as a semi-quantitative outcome measure, SUV may be affected by radiometabolites, blood flow, and clearance over time. Kinetic modelling with volume of distribution (*V*_T_) as an outcome measure accounts for these factors and provides a more robust assessment. Recent research using [^11^C]DPA713 PET/CT has shown the potential to quantify neuroinflammation in patients with painful cervical radiculopathy [26]. The [^11^C]DPA713 *V*_T_ was elevated in the affected spinal cord and neuroforamina compared with an unaffected tissue [26]. However, it remains unclear whether *V*_T_ is elevated in patients with painful cervical radiculopathy compared with pain-free participants and whether this relates to clinical parameters. Therefore, the primary objectives of this study were to (1) assess whether uptake of [^11^C]DPA713 in the spinal cord and neuroforamina is elevated in patients with painful cervical radiculopathy compared with pain-free participants (control group) and (2) assess whether [^11^C]DPA713 uptake is associated with clinical parameters, such as pain intensity, symptoms of depression, and disability. Based on preclinical CCI and CCD models [9,10,11,12,13], as well as previous human studies on lumbar radiculopathy and persistent pain [24,27], increased binding at the spinal cord and neuroforamina was anticipated in patients with painful cervical radiculopathy as compared with pain-free participants with associations to clinical parameters.

## 2. Methods

This study adhered to the Strengthening the Reporting of Observational Studies in Epidemiology (STROBE) guidelines [28]. Approval was obtained from the Medical Ethics Committee of Amsterdam University Medical Centre, location VUmc (Approval number: 2020.179), and was registered at the WHO International Clinical Trials Registry Platform (https://trialsearch.who.int; study ID: NL8060). Informed written consent was obtained from all participants prior to inclusion in the study.

### 2.1. Participants

People with painful cervical radiculopathy who meet the following diagnostic criteria were eligible: The diagnosis of painful cervical radiculopathy was made by a medical specialist based on the clinical diagnosis combined with the presence of confirmed nerve compression by Magnetic Resonance Imaging (MRI) (i.e., a relevant level of nerve compression had to match clinical parameters, which is considered the golden standard for diagnosing painful cervical radiculopathy [29]). Pain-free participants should not have had a painful condition in the preceding three months. Exclusion criteria were previous cumulated exposure to levels (>5 mSv) of radioactivity in the preceding year, immunosuppressive medication use, recent cervical epidural steroid injection, pregnancy, breastfeeding, and low-affinity TSPO polymorphism. TSPO polymorphism (rs6971) was determined using a PCR-based assay [26,30]. People with TSPO polymorphism were classified as A/A (high-affinity binders), whereas the others were either (A/G) (mixed affinity binders) or lacked polymorphism (G/G) (low-affinity binders).

### 2.2. Clinical Assessment, Physical Examination, and Questionnaires

Demographic and clinical data were obtained to describe participant characteristics and/or to assess the association between neuroinflammation and clinical parameters, such as arm pain and neck pain intensity (0–100 Visual Analogue Scale (VAS)). Physical tests, i.e., cervical range of rotation (CROM), pressure pain threshold (PPT), muscle strength (MRC muscle scale), reflexes, and sensory assessments, were performed. Questionnaires assessed disability (NDI), physical activity (IPAQ), the likelihood of central sensitisation (CSI), the likelihood of neuropathic pain (pain-DETECT), kinesiophobia (TSK-11), and symptoms of depression, anxiety, and stress (DASS21). Sleep quality was evaluated using the Pittsburgh Sleep Quality Index (PSQI). One clot-activated sample of peripheral blood (7 mL) was obtained by venipuncture from the antecubital fossa. Aliquots of blood samples to determine serum levels of high-sensitive c-reactive protein (hsCRP) were stored at −80 °C after centrifugation for 10 min at 1530× *g* at 21 °C. Serum levels of high-sensitivity C-reactive protein (hsCRP) were measured using the Cardiac C-Reactive Protein (Latex Slide Agglutination) High Sensitivity assay on Roche/Hitachi cobas c systems. Table 1 shows the clinical assessments and questionnaires used.

### 2.3. Scanning Protocol, Image Segmentation, and Kinetic Analysis

Participants underwent scanning on an Ingenuity TF PET/CT scanner (Philips Medical Systems, Best, The Netherlands). They were positioned within the axial field of view (18.4 cm) to encompass the affected neuroforamina (containing dorsal root ganglion and nerve roots), spinal cord, and the ascending aorta. The scanning protocol included a low-dose CT scan for attenuation correction and anatomical positioning. This was followed by a 60-min dynamic PET scan [46,47,48]. At the start of this scan, patients with cervical radiculopathy received an intravenous injection of 370 ± 22 MBq [^11^C]DPA713, and pain-free participants received 379 ± 18.7 MBq [11]DPA713. Among the four regularly used TSPO tracers—[^11^C]PK11195, [^11^C]PBR28, [^11^C]DPA713, and [^11^C]ER176, [^11^C]DPA713 was selected based on its superior signal-to-noise ratio, clinical applicability, and previous positive experiences in people with musculoskeletal pain within our hospital [25,48,49]. Radiotracer [^11^C]DPA713 was synthesized with high radiochemical purity [26]. All scans were reconstructed according to previously used protocols [26]. In short, list mode data were rebinned into 19 frames and reconstructed using the 3D RAMLA algorithm using CT-based attenuation correction. Final images had a voxel size of 4 mm^3^ and a spatial resolution of 5 mm full width at half maximum. Venous samples were taken to estimate the fraction of parent tracer at various time points. Image segmentation involved manual drawing of regions of interest (ROI) on axial CT slides, focusing on the affected neuroforamina (containing the dorsal root ganglion and nerve root), spinal cord, and ascending aorta, using ACCURATE softwarev01072023 [50,51]. Target ROIs were drawn using a circle with a diameter of 2 cm (one slice; volume: 1.5175 cm^3^). The center of this circle was placed at the center of the spinal canal, representing the spinal cord, and at the center of the neuroforamina, representing the neuroforamina. These regions were projected onto dynamic [^11^C]DPA713 images to extract regional time activity curves (TACs). Visual inspection of the scan, tissue time-activity curves over the 60 min scans, and fitting results were used to determine whether motion had affected the outcomes. Image-derived input curves were generated using a step-by-step process: (1) correcting for plasma-to-whole blood ratios, metabolite fractions, and scaling using early sample data; (2) replacing the curve tail with a multi-exponential fit; (3) adjusting for metabolites in plasma samples; and (4) correcting for delay in each TAC [52]. Given the challenges of determining radiometabolites at a later timepoint due to ^11^C decay, an additional analysis was performed using a plasma input model without metabolite correction (1T2k_WB) [26]. Tissue TACs were fitted to the 1T2k model, providing the *V*_T_ as the primary outcome measure. Secondary outcomes were other metrics of neuroinflammation, such as *V*_T_ derived from 1T2k_WB and Logan *V*_T_. The [^11^C]DPA713 was semi-quantified using the Logan *V*_T_ t* = 30 (Linearised method). Previously, we found that the 1T2k model is the optimal model to derive kinetic parameters in people with cervical radiculopathy at the neuroforamina and spinal cord, with high correlations with Logan *V*_T_ t* = 30 [26]. Logan plot analysis was used to generate parametric *V*_T_ images.

### 2.4. Sample Size

Based on linear regression, an α of 0.05, a β of 0.8, an expected effect size (d ≈ 0.9) [24]*,* and TSPO genotype as a covariate, a sample size of 6 in each group was required.

### 2.5. Statistical Analysis

Statistical analyses included linear regression to detect differences in [^11^C]DPA713 binding, using primary and secondary neuroinflammatory metrics (primary neuroinflammatory outcome: 1T2k *V*_T_; secondary neuroinflammatory outcomes: 1T2k_WB derived *V*_T_ and Logan *V*_T_) between people with painful cervical radiculopathy and pain-free participants. In this proof-of-concept study, Cohen’s d is employed to gauge the effect size. A value of 0.2 or less is considered a small effect, a value between 0.2 and 0.5 as a medium effect size, and a value of 0.8 or larger as a large effect [53]. Thereafter, associations between the different neuroinflammation metrics and clinical parameters were assessed using linear regression analysis and expressed as standardized beta. Because the rs6971 genotype might influence the binding of the [^11^C]DPA713 radiotracer, genotype was regarded as a confounding factor. Other potential confounding factors, such as symptom duration and medication use, were considered. We controlled for these factors through our selection criteria. Statistical significance was set at *p* < 0.05 using SPSS version 28.0 (IBM Corp, Armonk, NY, USA).

## 3. Results

### 3.1. Participants

Fifteen patients diagnosed with painful cervical radiculopathy and six pain-free participants were included in the study. Patients with painful cervical radiculopathy were also included in a separate study with identical selection criteria, which examined the effects of conservative intervention on spinal and neuroforaminal neuroinflammation. That study involved 15 patients with cervical radiculopathy. Although a power calculation showed that only 6 participants were needed, we opted to include all 15 in the present study. The C7 nerve root level was the most commonly affected (9/15, 60%), followed by the C6 nerve root (5/15, 33.3%), and C5 nerve root (1/15, 6.7%). Patients with painful cervical radiculopathy exhibited a higher prevalence and intensity of various clinical parameters when compared with their pain-free counterparts. Specifically, they experienced higher levels of neck pain, arm pain, neck pain disability, fear of movement, the likelihood of neuropathic pain, reduced physical activity levels, increased positive neurodynamic testing, diminished reflexes, heightened muscle weakness, greater sensory (vital/gnostic) loss, and more pain during maximal left and right cervical range of rotation. Table 2 provides an overview of baseline participant demographics, clinical characteristics, and functional profiles.

### 3.2. Levels of Neuroinflammation in Patients with Cervical Radiculopathy Compared with Pain-Free Participants

**Figure 1:**
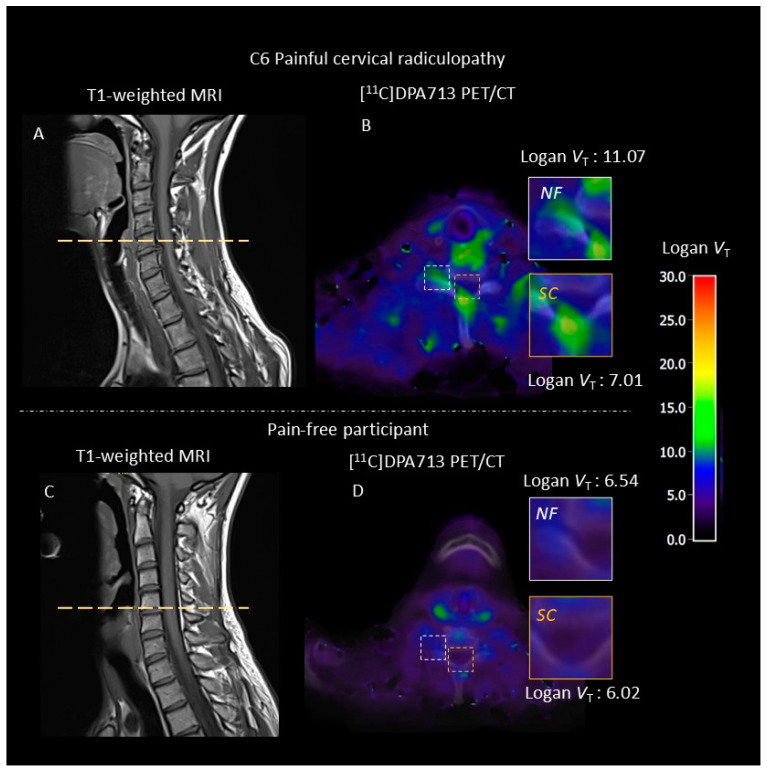
Neuroinflammation in a patient with a C6 right painful cervical radiculopathy and a pain-free participant. To quantify [^11^C]DPA713 binding at the neuroforamina and spinal cord, we utilized an image-derived input single tissue compartmental (1T2k) model. For visualization purposes, we generated volume of distribution (*V*_T_) images using Logan plot analysis, with a time threshold of t* = 30. (**A**): T1-weighted MRI of the patient with a painful cervical radiculopathy. The dotted line indicates the cross-sectional area used for the CT and PET analysis. (**B**): Parametric cross-sectional image of [^11^C]DPA713 binding merged with CT, with a zoomed view at the neuroforamina (NF) and spinal cord (SC). Higher volume of distribution (*V*_T_) indicates more tracer binding, suggesting higher levels of neuroinflammation. (**C**): T1-weighted MRI of a pain-free participant. (**D**): Follow-up parametric cross-sectional image of [^11^C]DPA713 binding merged with CT, with a zoomed view at the neuroforamina (NF) and spinal cord (SC).

Visual inspection of the scans indicated that no motion correction was needed. [^11^C]DPA713 *V*_T_ was significantly elevated in patients with painful cervical radiculopathy compared with pain-free participants at the neuroforamina (mean difference 4.74; Cohen’s d: 4.34; *p* = 0.04), but not at the spinal cord (mean difference 4.59, Cohen’s d: 6.36; *p* = 0.16). The 1T2K_WB derived *V*_T_ indicated significant elevated [^11^C]DPA713 binding in patients with cervical radiculopathy compared to pain-free participants at the neuroforamina (mean difference 3.74; Cohen’s d: 3.07; *p* = 0.02), but not at the spinal cord (mean difference 1.91, Cohen’s d: 5.06; *p* = 0.36). The linearized (Logan *V*_T_ t* = 30) demonstrated elevated radioligand binding at the neuroforamina (*p* = 0.03), but not at the spinal cord (*p* = 0.28). Table 3 and Table 4 present a comprehensive overview of the neuroinflammation metrics at the neuroforamina and spinal cord. Figure 1 shows typical parametric *V*_T_ images for a patient with a painful cervical radiculopathy and a pain-free participant.

### 3.3. Association Between Neuroinflammation and Clinical Parameters

**Figure 2:**
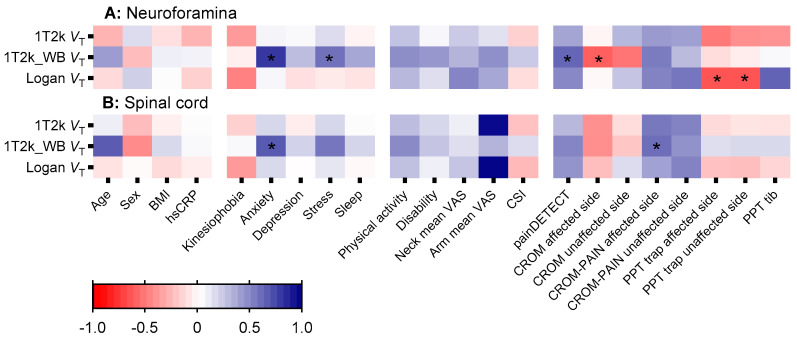
Heatmap showing the association (standardized-β) between non-linearized and linearized (semi-)quantification of [^11^C]DPA713 binding at the neuroforamina and spinal cord with clinical parameters in patients with cervical radiculopathy. * represents an association with a *p*-value below 0.05. No post-hoc comparisons were made. Associations were determined with genotype as a confounding factor. Abbreviations: 1T2k: single tissue compartmental model; 1T2k_WB: 1T2k without metabolite correction; BMI: Body Mass Index; hsCRP: high sensitive c-reactive protein; VAS: visual analogue scale; CSI: likelihood of central sensitization; painDETECT: likelihood of neuropathic pain; CROM-PAIN: Pain intensity at maximal cervical rotation Scale; CROM: maximal cervical rotation; PPT: Pressure Pain Threshold.

All association analyses were performed with genotype as a confounding variable and expressed as standardized coefficient β. Neuroforamina and spinal cord *V*_T_ did not reveal significant associations with clinical parameters in patients with cervical radiculopathy (Appendix A Table A1 and Table A2). Neuroforamina 1T2k_WB derived *V*_T_ was associated with symptoms scores of anxiety (β = 0.77; *p* = 0.01), psychological stress (β = 0.56; *p* = 0.04), maximal cervical range of rotation towards the affected side (β = −0.75; *p* = 0.01), and likelihood of neuropathic pain (β = 0.60; *p* = 0.04), while spinal cord 1T2k_WB derived *V*_T_ was associated with anxiety (β = 0.65; *p* = 0.01) and maximal cervical range of rotation towards the affected side (β = 0.57, *p* = 0.04). Neuroforamina Logan *V*_T_ found negative associations with trapezius muscle pressure pain threshold at the affected (β = −0.67; *p* = 0.02) and unaffected side (β = −0.67; *p* = 0.02), with no associations between spinal cord Logan *V*_T_ and clinical parameters. Figure 2 provides a heatmap depicting the associations between various neuroinflammation metrics and clinical parameters for the neuroforamina and spinal cord.

## 4. Discussion

The key findings of this study are summarized into three main points. This is the first study to demonstrate that patients with painful cervical radiculopathy display higher levels of neuroinflammation at the affected neuroforamina, but not at the spinal cord, relative to pain-free participants. Secondly, neuroforamina and spinal cord neuroinflammation, quantified using [^11^C]DPA713 *V*_T_, are not associated with clinical parameters. Thirdly, secondary neuroforamina and spinal cord neuroinflammation metrics revealed associations with clinical parameters such as kinesiophobia, symptoms of depression, physical activity, likelihood of neuropathic pain, cervical range of motion, pain intensity at maximal cervical range of motion, and pressure pain thresholds.

### 4.1. Comparison with Existing Literature

Several preclinical studies have provided strong evidence for the involvement of DRG and spinal cord neuroinflammation in the development of chronic and neuropathic pain [7,14,16,17,54]. In a preliminary investigation, we identified the optimal pharmacokinetic model to describe the uptake of [^11^C]DPA713 at the neuroforamina and spinal cord in patients with painful cervical radiculopathy [26]. Our findings indicated that the single-tissue compartmental model was the most suitable non-linearized model [26]. Additionally, we observed a high correlation between Logan *V*_T_ with 1T2k derived *V*_T_, suggesting that Logan *V*_T_ could serve as viable options for semi-quantifying the uptake of [^11^C]-DPA713 [26]. In that study, we detected elevated neuroforamina and spinal cord neuroinflammation compared with unaffected tissue, demonstrating the presence of neuroinflammation at the affected tissues compared with unaffected tissues in people with painful cervical radiculopathy [26]. However, another study examining [^11^C]DPA713 binding at the spinal cord in patients with cervical radiculopathy found no elevations in standardized uptake values (SUV) compared with pain-free participants [25]. We strengthened these results as we also did not find elevated spinal cord neuroinflammation using 1T2k *V*_T_ and our secondary neuroinflammatory metrics.

The use of 1T2k-derived V_T_ as a binding metric represents an advancement in the field, as simplified measures like SUV do not account for factors such as blood flow, radiometabolites, and blood clearance over time. However, V_T_ does not correct for non-specific binding, the amount of non-specific binding could be assessed with a study in the same subject with and without an additional target blocking agent targeting TSPO or a validated reference region to calculate non-displaceable binding. Nonetheless, as no reference region free of specific binding is known for the spinal region, applying a reference tissue approach also has its limitations [55]. Comparing our results with other neuropathic pain conditions reveals that patients with lumbar radiculopathy exhibited increased SUV and SUV ratio levels at the neuroforamina and spinal cord [24]. In contrast, we were unable to detect significant spinal cord neuroinflammation, which may be attributed to differences in neuropathy severity. It should be noted that we used [^11^C]DPA713 as a tracer, while in the other study [^11^C]PBR28 was used. Nevertheless, both tracers have a high affinity for TSPO and a favorable specific binding to nonspecific binding ratio [24,49]. To support the notion that remote immune-mediated inflammation is associated with the severity of neuroinflammation, a previous study has shown that patients with severe traumatic nerve injury exhibit elevated V_T_ in the thalamus [56]. It is highly plausible that the mild compression associated with painful cervical radiculopathy may not be severe enough to induce spinal cord neuroinflammation in patients with painful cervical radiculopathy [19]. The injury of a mild compression neuropathy is probably not severe enough to cause neuroinflammation at the spinal cord in humans, in contrast to experimental animal models of neuropathic pain [11,19,21]. Translating these findings on immune-mediated neuroinflammation into clinical applications remains an important next step [27].

### 4.2. Clinical Considerations

Targeting glial cells, such as microglia and astrocytes, and/or macrophage-mediated neuroinflammatory responses has demonstrated efficacy in preventing or reversing the establishment of persistent pain behaviors in preclinical studies of neuropathic pain (e.g., treatment by physical exercise, nerve mobilization, or minocycline) [14,57,58]. The present study did not find meaningful associations between [^11^C]DPA713 *V*_T_ (primary neuroinflammatory metric) and clinical parameters such as pain intensity or disability levels. One explanation might be the low variance in our small sample for pain intensity and disability. Another reason might be that pain intensity is considered to be the cortical encoding of nociception, but that other biopsychosocial factors might disrupt the association between neuroinflammation and pain intensity. However, due to the small number of participants, we are unable to test this hypothesis. It is more plausible that metabolite correction induced variability in *V*_T_, resulting in the loss of its association with clinical parameters. A second image-derived input was created without correcting for metabolites, as previous research has shown that metabolite correction might induce additional variability and is not superior to non-corrected metabolite analysis [26]. The 1T2k_WB derived *V*_T_ revealed associations with clinical outcomes such as the likelihood of neuropathic pain, potentially indicating that the metabolite correction causes the loss in clinical parameter associations.

Another study conducted in patients with painful lumbar radiculopathy revealed elevated neuroinflammation (SUV ratio using the [^11^C]PBR28 radiotracer; contralateral neuroforamina and thoracic spinal cord served as unaffected reference tissues) at the lumbar neuroforamina and spinal cord, which correlated with responses to epidural fluoroscopy-guided steroid injection [24]. While the effectiveness of cervical epidural steroid injection remains uncertain, with some studies reporting positive effects while others do not [59,60], patients with high levels of [^11^C]DPA713 binding at the neuroforamina might potentially benefit from this treatment approach [61]. Targeting the individual pathophysiology in patients with painful cervical radiculopathy and matching this with a treatment known for targeting that specific pathophysiology could lead to more personalized and effective treatment. As we tested for elevated neuroinflammation at the neuroforamina between patients with painful cervical radiculopathy and pain-free participants, the box-plot in Figure 2A revealed that approximately 50% of all patients with painful cervical radiculopathy exhibit higher neuroinflammation levels compared to the highest level in the pain-free participants. This indicates that neuroforaminal neuroinflammation is highly variable among patients with painful cervical radiculopathy. Further research is needed to establish cut-off values for detecting elevated neuroforaminal neuroinflammation. However, given the high clinical costs, limited availability, and patient burden, PET imaging should not be used as a routine diagnostic tool [27]. Comparative studies between systemic proteomics and PET imaging could offer valuable insights into potential blood-based biomarkers as alternative diagnostic tools.

(Pre)clinical research showed that conservative treatments, such as physical exercise [62] and/or neural mobilization [63], have an effect on neuroinflammation in animal models with induced compression neuropathies. Therefore, these treatments may be suitable to decrease neuroinflammation and improve clinical recovery. A limitation of cross-sectional studies is that no cause-effect associations can be established. By conducting clinical studies that target the aberrant neuroinflammation in patients with painful cervical radiculopathy in association with clinical parameters, the cause-effect relationship between neuroinflammation and clinical parameters can be investigated.

### 4.3. Limitations and Further Research

Several considerations must be taken into account when interpreting our findings. Firstly, 1T2k *V*_T_ is the primary outcome to measure neuroinflammation at the neuroforamina and spinal cord in patients with painful cervical radiculopathy [26]. While other neuroinflammation metrics, such as Logan *V*_T,_ show strong correlations with 1T2k *V*_T_, these may introduce additional bias (e.g., biased estimates as they may not capture all nuances of tracer kinetics) [26]. Secondly, as our study serves as a proof-of-principle study, the initial sample size was relatively small. Further studies are imperative to replicate our findings in larger cohorts. Due to the invasive nature of dynamic PET/CT imaging, its associated radiation exposure, and the physical demands on participants—such as remaining still for 60 min—this imaging technique is typically reserved for more severe patient groups like neurodegenerative diseases and cancer. It is hardly employed for patient groups, such as those with cervical radiculopathy. Ethical constraints therefore limit the number of participants that can be included in such studies. Nevertheless, with the number of participants included, we successfully confirmed the hypothesis of increased neuroforaminal inflammation in patients with cervical radiculopathy.

Moreover, *V*_T_ was assessed using non-linear kinetic modeling with metabolite correction as the primary outcome measure. Prior research demonstrates that the 1T2k pharmacokinetic model best describes radiotracer uptake [22]. However, given the challenges of measuring radiometabolites at low concentrations, we also provide *V*_T_ results derived from 1T2k modeling without metabolite correction and from linear Logan modeling. All three approaches yielded consistent findings, confirming elevated radiotracer uptake at the neuroforamina in patients with cervical radiculopathy.

We did not detect an elevated PET signal in the spinal cord in the patients with painful cervical radiculopathy compared to the pain-free participants. In our analysis, partial volume correction (PVC) was not applied, as it was not feasible on the used scanner, and alternative software solutions are not robust enough, introducing additional uncertainty. Therefore, future studies should use long axial field-of-view scanners to improve sensitivity and PVC correction. The arrival of long-axial field-of-view PET/CT scanners offers higher precision in measuring neuroinflammation [64]. The advantage of using long-axial field-of-view PET/CT scans lies in capturing the entire neuroaxis in the field of view, enabling imaging not only of the affected neuroforamina and spinal cord but also of supraspinal regions, such as the thalamus and cingulate cortices, where neuroinflammation may also be manifest [65,66,67,68]. However, this supraspinal neuroinflammation might not be a direct consequence of mild nerve compression but is more likely the result of pain-comorbid negative affect [66].

## Figures and Tables

**Table 1:** Description of clinical assessment and questionnaires used.

Clinical Assessment	Physical Examination and Questionnaire	Description
Pain intensity	Visual analogue scale (VAS)	The VAS for pain is a commonly used assessment tool in healthcare to measure a patient’s pain intensity. It consists of a straight line, typically 10 centimetres in length, with “no pain” at one end and “worst pain imaginable” at the other. Participants are asked to mark the point on the line that corresponds to the intensity of their pain, providing a subjective but quantifiable measure of their discomfort. The mean pain intensity of the last 24 h was recorded.
Neck disability	Neck disability questionnaire (NDI)	The NDI consists of ten items: pain intensity, personal care, lifting, reading, headaches, concentration, work, driving, sleeping, and recreation. Each item has six different assertions expressing progressive levels of pain or limitation in activities. Item scores range from 0 (no pain or limitation) to 5 (as much pain as possible or maximal limitation). The total NDI score ranges from 0 to 50 points. Higher scores indicate greater disability. The Dutch version of the NDI has been shown to be a valid and responsive measure of disability [31].
Likelihood of neuropathic pain	painDETECT (PD-Q)	The PD-Q is a simple tool to predict the likelihood of a neuropathic pain component being present in persistent pain patients [32]. The persistent pain will be categorized into two-mechanism based groups: nociceptive and neuropathic using the PD-Q [33]. The PD-Q is a reliable screening tool with high sensitivity and specificity [32]. The questionnaire consists of 7 questions regarding the graduation of pain, pain course pattern, and radiating pain. The result score of the painDETECT will be used on a continuous scale.
Likelihood of Central Sensitisation	Central Sensitisation Inventory (CSI)	The CSI is a self-report screening instrument to help identify patients with central sensitivity syndromes. A cut-off score of 40 out of 100 best distinguishes between central sensitivity disorders and a non-patient comparison with a sensitivity of 81% and specificity of 75% [34]. The Dutch Central Sensitization Inventory (CSI) questionnaire has good internal consistency, good discriminative power, and excellent test-retest reliability [35]. The result score of the CSI will be used on a continuous scale.
Physical activity	International Physical Activity Questionnaire—short form (IPAQ)	The IPAQ is an internationally recognized questionnaire to measure physical activity [36]. The short form of the IPAQ consists of seven items that are used to estimate the total amount of physical activity expressed in metabolic equivalent minutes per week and time spent sitting [37].
Sleep quality	Pittsburg Sleep Quality Index (PSQI)	The PSQI measures subjective sleep quality [38] and is frequently used in pain research [39,40].
Depressive, anxiety, and stress symptoms	Depression, Anxiety, Stress Scale (DASS21)	The DASS21 is the preferred questionnaire to assess depression, anxiety, stress, and fear of movement in musculoskeletal pain [41,42].
Kinesiophobia	Tampa Scale for Kinesiophobia (TSK-11)	The TSK-11 assess fear of movement- related pain and had an acceptable to excellent inter-consistency and high test-retest reliability [43]. To evaluate fear of movement in individuals with musculoskeletal pain, the TSK-11 is recommended [41].
Myotome	n/a	C4: shoulder elevation; C5 Shoulder abduction; C6 flexion elbow; C7 Extension elbow; C8: wrist extension; Th1: adduction thumb/ spreading fingers. Muscle strength was scored using the Medical Research Council (MRC 0–5) scale for muscle strength. It was recorded if a patient had an MRC score less than normal (5 = normal).
Reflexes	n/a	Biceps brachii, triceps brachii reflexes. It was recorded if a patient had a hyporeflexia graded on a scale of −4 (absent) to +4 (continuous clonus)
Gnostic and vital sensibility	n/a	The gnostic sensibility was assessed by gently brushing the skin area, while vital sensibility was evaluated with the use of a sharp skin roller. Any loss of sensation according to a dermatomal pattern in a participant was documented.
Upper Limb Tension Test	ULTT1	The ULTT1 is designed to stress the median nerve, the anterior interosseous nerve, and the nerve roots of C5 to C7. It involves positioning the patient with their affected arm abducted, wrist and fingers extended, while the examiner laterally flexes the patient’s neck to the opposite side, looking for any reproduction of symptoms along the nerve pathway[44].
Pressure Pain Threshold	PPT	The pressure pain threshold is defined as the amount of pressure required for the pressure sensation to first change to pain[45]. A baseline algometry was used to measure PPT levels. The electronic algometer (Somedic AB, FArsta, Sweden) consists of a 1-cm^2^ rubber, approximally 50 kPa/s increase in force was given. Subjects are instructed to press a button attached to the algometer when the sensation changed from pressure to pain. The mean of 3 trials was calculated and used for analysis. A 30-s resting period will be allowed between each measure. To determine changes in widespread pressure pain sensitivity, PPTs are assessed bilaterally over the mid-point trapezius muscle (pars descendens), and the non-dominant tibialis anterior muscle.
Systemic inflammation	Serum high sensitive c-reactive protein	Serum levels of high-sensitive CRP (hsCRP) were measured using Cardiac C-Reactive Protein (Latex Slide Agglutination) High Sensitivity using Roche/Hitachi cobas c systems. Because of the heightened sensitivity of hsCRP (with a lower limit of quantification of 0.3 mg/L) in comparison to CRP (with a lower limit of quantification of 0.6 mg/L), and anticipating very low hsCRP/CRP levels, we chose to utilise the hsCRP assay over the CRP assay.

**Table 2:** Overview of the baseline participant demographics, clinical characteristics, and functional profiles.

	Patients with Painful Radiculopathy (*n* = 15) Mean (SD)	Pain-Free Participants (*n* = 6) Mean (SD)	*p*-Value
**Participant demographics**			
Age (Years)	50 (12)	43 (12)	0.22
Duration of symptoms (weeks)	49 (21)	N/A	N/A
Sex (% male)	53	50	0.89
BMI	25 (3.5)	25 (1.8)	0.94
Systemic hsCRP (mg/L) (median, 25th–75th percentile)	1.04 (0.55–2.76)	0.51 (0.15–0.76)	0.05
TSPO genotype (% high affinity)	46%	50%	0.89
Injected dose, mCi	370 (22)	379 (18.7)	0.28
**Questionnaires**			
Physical activity level (IPAQ)	736 (800)	2583 (1760)	0.003
Neck pain intensity (mean VAS, 0–100)	47 (22)	0 (0)	N/A
Arm pain intensity (mean VAS, 0–100)	54 (23)	0 (0)	N/A
Likelihood of neuropathic pain (pain_DETECT, 0–30)	16 (6.5)	4 (6.9)	0.001
Likelihood of central sensitisation (CSI, 0–100)	39 (20)	22 (11)	0.06
Neck disability (NDI, 0–50)	20 (9.2)	3 (3.8)	<0.001
Kinesiophobia (TSK-11, 0–44)	28 (7.1)	13 (2.2)	<0.001
Sleep quality (PSQI, 0–21)	18 (7.9)	10 (7.1)	0.06
Psychological stress (DASS21, 0–21)	4.5 (4.2)	4.3 (5.3)	0.95
Anxiety (DASS21, 0–21)	2.5 (3.7)	1.3 (1.2)	0.46
Depression (DASS21, 0–21)	4 (5)	2 (4)	0.47
**Physical examination**			
ULTT1 positive	80%	0%	N/A
Reduced reflexes	100%	0%	N/A
Muscle weakness	100%	0%	N/A
Vital sensory changes	100%	0%	N/A
Gnostic sensory changes	100%	0%	N/A
PPT trapezius affected side	353 (180)	486 (93)	0.10
PPT trapezius unaffected side	355 (225)	458 (97)	0.33
PPT tibialis anterior	348 (166)	678 (172)	0.02
Cervical rotation affected side	64 (15)	74 (13)	0.15
Pain intensity at maximal cervical rotation affected side	34 (30)	0 (0)	N/A
Cervical rotation unaffected side	64 (18)	72 (14)	0.34
Pain intensity at maximal cervical rotation unaffected side	34 (30)	0 (0)	N/A

Abbreviations: TSPO: Translocater protein, BMI: Body Mass Index, ULLT1: Upper Limb Tension Test for nerve roots C5 to C7, PPT: Pressure Pain Threshold, CRP: high sensitive c-reactive protein, N/A: non applicable.

**Table 3:** Overview showing non-linearized and linearized semi-quantification of [^11^C]DPA713 binding at the neuroforamina for the patients with painful cervical radiculopathy and pain-free participants.

Neuroforamina	Painful Cervical Radiculopathy Mean (SD)	Pain-Free Participants Mean (SD)	Mean Difference (SE)	Cohen’s d	*p*-Value
*V*_T_ 1T2k	14.19 (4.78)	9.44 (3.16)	4.74 (2.17)	4.34	0.04
*V*_T_ 1T2k_WB	10.53 (3.41)	6.78 (1.94)	3.74 (1.50)	3.07	0.02
*V*_T_ Logan	12.31 (5.74)	7.30 (1.08)	5.01 (2.39)	4.86	0.03

Abbreviations: 1T2k: single tissue compartmental model; 1T2k_WB: 1T2k model without metabolite correction.

**Table 4:** Overview showing non-linearized and linearized semi-quantification of [^11^C]DPA713 binding at the spinal cord for the patients with cervical radiculopathy and pain-free participants.

Spinal Cord	Painful Cervical Radiculopathy Mean (SD)	Pain-Free Participants Mean (SD)	Mean Difference (SE)	Cohen’s d	*p*-Value
*V*_T_ 1T2k	14.28 (7.13)	9.69 (3.97)	4.59 (3.14)	6.36	0.16
*V*_T_ 1T2k_WB	10.39 (5.18)	8.48 (4.68)	1.91 (2.44)	5.06	0.36
*V*_T_ Logan	12.37 (5.67)	9.25 (3.77)	3.12 (2.77)	5.27	0.28

Abbreviations: 1T2k: single tissue compartmental model;1T2k_WB: 1T2k without metabolite correction.

## Data Availability

Individual deidentified participant data that underlie the results will be shared. Investigators whose proposed use of the data had been approved by an independent review committee identified for this purpose can access the data for individual participant data meta-analysis. Data will be available beginning 9 months and ending 36 months following article publication. Proposals may be submitted up to 36 months following article publication. After 36 months the data will be available in our University’s data warehouse but without investigator support other than deposited metadata. Information regarding submitting proposals and accessing data may be found at https://research.vu.nl.

## Data Collection

Data were collected between November 2021 and April 2023.

## Appendix A

**Table A1:** Shows the association between non-linearised and linearised semi-quantification of [^11^C]DPA713 binding at the neuroforamina with clinical parameters in patients with painful cervical radiculopathy.

Neuroforamina	1T2k *V*_T_	*p*-Value	1T2k_WB *V*_T_	*p*-Value	Logan *V*_T_	*p* Value
Age	−0.30 (0.13)	0.39	0.39 (0.09)	0.20	−0.15 (0.15)	0.65
Sex	0.14 (3.11)	0.69	−0.27 (1.98)	0.38	0.19 (3.49)	0.54
BMI	−0.30 (0.45)	0.37	0.06 (0.29)	0.85	−0.19 (0.54)	0.54
hsCRP (mg/L)	−0.13 (0.14)	0.71	0.05 (0.010	0.89	0.01 (0.17)	0.98
Physical Activity (IPAQ)	0.24 (0.01)	0.49	0.45 (0.00)	0.12	0.28 (0.01)	0.37
Kinesiophobia (TSK-11, 0–44)	−0.40 (0.19)	0.22	−0.07 (0.14)	0.81	−0.50 (0.20)	0.09
Anxiety (DASS21, 0–21)	0.04 (0.40)	0.91	0.77 (0.19)	0.01	0.03 (0.45)	0.94
Depression (DASS21, 0–21)	0.02 (0.28)	0.96	0.26 (0.19)	0.37	−0.13 (0.32)	0.67
Stress (DASS21, 0–21)	0.14 (0.40)	0.68	0.56 (0.21)	0.04	−0.09 (0.44)	0.77
Sleep (PSQI, 0–21)	−0.05 (0.19)	0.89	0.35 (0.12)	0.24	−0.12 (0.21)	0.69
Neck pain intensity (VAS 0–100)	0.21 (0.08)	0.52	0.30 (0.04)	0.34	0.48 (0.08)	0.11
Arm pain intensity (VAS 0–100)	0.12 (0.07)	0.75	0.44 (0.00)	0.87	0.35 (0.07)	0.29
Likelihood of central sensitisation (CSI, 0–100)	−0.19 (0.09)	0.56	0.21 (0.05)	0.49	−0.14 (0.10)	0.66
Likelihood of neuropathic pain (PainDetect, 0–30)	0.36 (0.23)	0.30	0.60 (0.14)	0.04	0.47 (0.25)	0.14
Cervical range of rotation affected side	−0.04 (0.12)	0.91	−0.63 (0.07)	0.04	−0.03 (0.14)	0.93
Cervical range of rotation unaffected side	0.29 (0.10)	0.39	−0.55 (0.06)	0.10	0.23 (0.12)	0.46
Pain at maximal cervical rotation affected side	0.40 (0.06)	0.22	0.52 (0.03)	0.12	0.49 (0.06)	0.10
Pain at maximal cervical rotation VAS unaffected side	0.38 (0.05)	0.26	0.27 (0.04)	0.44	0.54 (0.05)	0.07
PPT trapezius affected side	−0.53 (0.01)	0.09	−0.14 (0.01)	0.65	−0.67 (0.01)	0.02
PPT trapezius unaffected side	−0.45 (0.01)	0.17	−0.08 (0.01)	0.81	−0.67 (0.01)	0.02
PPT tibialis anterior muscle	−0.43 (0.01)	0.19	0.02 (0.00)	0.94	0.61 (0.01)	0.03

Association determined using linear regression analysis with genotype as confounder. Results expressed as standardised beta with standard error. PPT: Pressure Pain Threshold; hsCRP: high-sensitive c-reactive protein; BMI: Body Mass Index.

**Table A2:** Shows the association between non-linearised and linearised semi-quantification of [^11^C]DPA713 binding at the spinal cord with clinical parameters in patients with cervical radiculopathy.

Spinal Cord	1T2k *V*_T_	*p*-Value	1T2k_WB *V*_T_	*p*-Value	Logan *V*_T_	*p* Value
Age	0.07 (0.19)	0.82	0.64 (0.13)	0.83	−0.12 (0.17)	0.71
Sex	−0.27 (4.23)	0.29	−0.46 (2.77)	0.12	−0.02 (3.85)	0.96
BMI	0.01 (0.67)	0.99	0.15 (0.43)	0.60	−0.08 (0.55)	0.81
hsCRP (mg/L)	−0.07 (0.21)	0.84	0.02 (0.15)	0.95	−0.15 (0.17)	0.66
Physical Activity (IPAQ)	−0.19 (−0.65)	0.53	0.02 (0.21)	0.95	−0.40 (0.22)	0.20
Kinesiophobia (TSK-11, 0–44)	0.16 (0.55)	0.63	0.65 (0.31)	0.01	0.15 (0.45)	0.64
Anxiety (DASS21, 0–21)	−0.08 (0.40)	0.79	0.18 (0.29)	0.55	−0.01 (0.62)	0.97
Depression (DASS21, 0–21)	0.16 (0.54)	0.61	0.54 (0.31)	0.05	0.10 (0.44)	0.76
Stress (DASS21, 0–21)	0.01 (0.26)	0.99	0.17 (0.19)	0.56	−0.03 (0.23)	0.93
Sleep (PSQI, 0–21)	0.26 (0.01)	0.40	0.46 (0.00)	0.10	0.25 (0.01)	0.44
Neck pain intensity (VAS 0–100)	0.16 (0.25)	0.63	0.23 (0.17)	0.45	0.06 (0.21)	0.85
Arm pain intensity (VAS 0–100)	0.07 (0.11)	0.82	0.13 (0.07)	0.66	0.23 (0.09)	0.47
Likelihood of central sensitisation (CSI, 0–100)	0.93 (0.00)	0.99	0.30 (0.32)	0.80	0.96 (0.11)	0.32
Likelihood of neuropathic pain (PainDetect, 0–30)	−0.25 (0.12)	0.41	0.14 (0.08)	0.63	−0.28 (0.09)	0.39
Cervical range of rotation affected side	0.28 (0.33)	0.38	0.48 (0.23)	0.12	0.42 (0.26)	0.21
Cervical range of rotation unaffected side	−0.44 (0.16)	0.17	−0.45 (0.10)	0.13	−0.26 (0.12)	0.43
Pain at maximal cervical rotation affected side	−0.14 (0.15)	0.65	−0.23 (0.10)	0.45	0.14 (0.12)	0.67
Pain at maximal cervical rotation VAS unaffected side	0.53 (0.07)	0.07	0.57 (0.05)	0.04	0.37 (0.06)	0.24
PPT trapezius affected side	0.49 (0.07)	0.09	0.43 (0.05)	0.14	0.49 (0.06)	0.10
PPT trapezius unaffected side	−0.15 (0.01)	0.64	0.13 (0.01)	0.68	−0.25 (0.01)	0.44
PPT tibialis anterior muscle	−0.11 (0.01)	0.73	0.15 (0.01)	0.63	−0.26 (0.01)	0.43
PPT tibialis anterior muscle	−0.12 (0.01)	0.70	0.15 (0.01)	0.63	−0.17 (0.01)	0.61

Association determined using linear regression analysis with genotype as confounder. Results expressed as standardised beta with standard error. PPT: Pressure Pain Threshold; hsCRP: high-sensitive c-reactive protein; BMI: Body Mass Index.
